# Targeted Therapies in Inflammatory Bowel Disease: Mechanisms, Comparative Evidence, and Clinical Translation

**DOI:** 10.3390/jcm15176753

**Published:** 2026-08-31

**Authors:** Christopher Pavel, Ana Popa, Madalina Ilie, Oana-Mihaela Plotogea, Raluca-Ioana Dascalu, Teodor Cabel, Deniz Gunsahin, Iulia Tincu, Bogdan Gaspar, Luiza Elena Pavel, Alexandra Oaie

**Affiliations:** 1 Faculty of Medicine, “Carol Davila” University of Medicine and Pharmacy, 050474 Bucharest, Romania; christopher.pavel@gmail.com (C.P.); plotogea.oana@gmail.com (O.-M.P.); raluca-ioana.dascalu@rez.umfcd.ro (R.-I.D.); teodor.cabel@drd.umfcd.ro (T.C.); iulia.tincu@umfcd.ro (I.T.); bogdan.gaspar@umfcd.ro (B.G.);; 2 Department of Gastroenterology, Clinical Emergency Hospital of Bucharest, 014461 Bucharest, Romania; anamaria98popa@gmail.com (A.P.); alexandra-anamaria.oaie@rez.umfcd.ro (A.O.); 3 Department of Pediatrics, “Dr. Victor Gomoiu” Children’s Clinical Hospital, 022102 Bucharest, Romania; 4 Department of Surgery, Clinical Emergency Hospital of Bucharest, 014461 Bucharest, Romania; 5 Department of Pathology, Emergency University Hospital, 050098 Bucharest, Romania

**Keywords:** inflammatory bowel disease, Crohn’s disease, ulcerative colitis, biological therapy, JAK inhibitors, IL-23 inhibitors, anti-TNF, vedolizumab, ustekinumab, TL1A, targeted therapy

## Abstract

Inflammatory bowel disease (IBD), encompassing Crohn’s disease (CD) and ulcerative colitis (UC), is a chronic immune-mediated condition with substantial global burden and rising incidence, requiring an expanding range of advanced therapies. This focused narrative review examines the mechanistic rationale, comparative clinical evidence, and translational implications of approved and emerging targeted therapies for adult IBD. A structured literature search was conducted in PubMed, Scopus, and Web of Science for publications from January 2021 to March 2026. Thirty-eight articles were selected as the core evidence set with landmark trials and clinical guidelines added when needed for historical or practice context. Evidence was synthesised narratively, with an explicit distinction between direct head-to-head comparisons, placebo-controlled trials, indirect network comparisons, and observational data. In UC, VARSITY showed higher week-52 clinical remission and endoscopic improvement with vedolizumab than adalimumab; in CD after anti–tumour necrosis factor (anti-TNF) failure, SEQUENCE showed risankizumab noninferior to ustekinumab for week-24 clinical remission and superior for week-48 endoscopic remission. Agents targeting tumour necrosis factor-like cytokine 1A (TL1A) have shown encouraging activity in phase 2/2b trials, but long-term effectiveness and safety remain uncertain. The current evidence supports mechanism-informed rather than biomarker-defined treatment selection. Confidence in comparative conclusions is greatest when supported by direct randomised evidence, whereas observational and indirect comparisons require caution because of confounding, heterogeneity, and differences in populations and outcome definitions. Clinical translation therefore requires the integration of the disease phenotype, prior treatment exposure, safety risks, and patient preference, with objective reassessment after therapy initiation.

## 1. Introduction

Inflammatory bowel disease (IBD) is a chronic, immune-mediated inflammatory condition of the gastrointestinal tract, encompassing two major clinical entities: Crohn’s disease (CD) and ulcerative colitis (UC). While UC is characterised by continuous mucosal inflammation confined to the colon and rectum, CD may affect any segment of the digestive tract in a transmural and discontinuous manner, giving rise to a wide spectrum of clinical phenotypes. Both conditions follow a relapsing-remitting course and are associated with significant morbidity, including intestinal complications such as strictures, fistulae, and abscesses, as well as extraintestinal manifestations and a markedly impaired quality of life [1]. Once regarded as a predominantly Western disease, IBD has progressively become a global health concern, with rising incidence now documented across Asia, South America, and the Middle East, driven by urbanisation and shifts in environmental exposures [2].

The conventional pharmacological approach to IBD has long been structured around aminosalicylates, corticosteroids, and immunomodulators such as azathioprine and methotrexate. Although these agents remain in clinical use, their limitations are well established: corticosteroids are unsuitable for long-term maintenance owing to cumulative toxicity, while immunomodulators are associated with a slow onset of action and a narrow therapeutic window. The introduction of anti-tumour necrosis factor (anti-TNF) agents—primarily infliximab and adalimumab—represented a pivotal advance, enabling mucosal healing and reducing the rates of hospitalisation and surgery [1]. However, the long-term effectiveness of anti-TNF therapy is constrained by primary non-response in a substantial minority of patients and by secondary loss of response driven by immunogenicity and subtherapeutic drug levels over time [3]. Data from the prospective PANTS cohort study confirmed that a significant proportion of patients fail to maintain remission beyond the first year of treatment, underscoring the need for alternative therapeutic strategies [3].

Progress in understanding the immunopathogenesis of IBD has revealed a complex network of inflammatory mediators and signalling cascades that drive intestinal injury. Key pathways implicated include the interleukin-12/interleukin-23 (IL-12/IL-23) and selective IL-23 axes, which orchestrate T-helper cell polarisation and mucosal inflammation; the Janus kinase–signal transducer and activator of transcription (JAK–STAT) signalling pathway, which mediates cytokine receptor transduction; and the sphingosine-1-phosphate (S1P) receptor system, which regulates lymphocyte trafficking from secondary lymphoid organs to the gut [2,4]. The identification of these pathways has catalysed the development of a new generation of biologic agents and small molecules. Integrin blockade with vedolizumab, IL-12/IL-23 inhibition with ustekinumab, selective IL-23 inhibition with risankizumab and mirikizumab, JAK inhibition with upadacitinib and filgotinib, and S1P receptor modulation with ozanimod and etrasimod now constitute a substantially expanded therapeutic arsenal [2,5,6].

Despite this remarkable expansion, the evidence base remains fragmented. Many recent publications focus on individual mechanisms, single drug classes, or disease-specific comparative rankings [4,7,8,9,10,11,12]. Direct head-to-head trials remain uncommon, and network meta-analyses, pivotal placebo-controlled trials, and real-world cohorts answer different clinical questions while carrying different risks of bias. Consequently, treatment rankings cannot be transferred uncritically across Crohn’s disease and ulcerative colitis, biologic-naive and biologic-experienced populations, or patients with different safety profiles [5,13,14,15]. A synthesis that connects mechanism, evidence design, and clinical translation is therefore needed. By bringing together the mechanistic rationale, direct and indirect comparative evidence, and practical considerations for treatment selection, this review aims to provide a clinically oriented synthesis that clearly distinguishes established therapies from emerging strategies targeting tumour necrosis factor-like cytokine 1A (TL1A). The objective of this focused narrative review is to integrate the mechanistic rationale, comparative efficacy and safety evidence, evidence maturity, and translational relevance of approved and emerging targeted therapies for adult IBD. Its distinguishing contribution is the explicit separation of direct from indirect comparative evidence, the critical interpretation of design-specific limitations, and the translation of these findings into a non-prescriptive framework for clinical decision-making and future research.

Throughout this review, we use the term “advanced therapies” to include both biologic agents and targeted small molecules. Biologic therapies are monoclonal antibodies directed against inflammatory cytokines or pathways involved in leukocyte trafficking. Targeted small molecules are oral agents that interfere with intracellular signalling or lymphocyte trafficking. Agents that are still under clinical investigation and have not been approved for routine use in IBD are referred to as “emerging therapies.”

## 2. Materials and Methods

### 2.1. Search Strategy

For this focused narrative review, a structured literature search was conducted across PubMed, Scopus, and Web of Science for publications from January 2021 to March 2026. The search combined the following Medical Subject Headings (MeSH) terms and free-text keywords: “inflammatory bowel disease”, “Crohn’s disease”, “ulcerative colitis”, “biological therapy”, “targeted therapy”, “biologic agents”, “JAK inhibitors”, “IL-23 inhibitors”, “anti-TNF”, “vedolizumab”, “ustekinumab”, “small molecules”, and “sphingosine-1-phosphate receptor modulators”. Boolean operators (AND, OR) were applied to optimise search sensitivity and specificity. No language restrictions were applied at the search stage. The search was designed to support a focused, clinically oriented synthesis rather than a formal systematic review or meta-analysis.

The final database search was conducted on 31 March 2026. The complete database-specific search strategies, including the Boolean combinations, search fields, and date limits, are provided in Supplementary Table S1. Records retrieved from the three databases were imported into Rayyan (Rayyan Systems Inc., Cambridge, MA, USA; https://www.rayyan.ai/; accessed on 31 March 2026). Potential duplicate records were identified using the platform’s duplicate-detection function and were subsequently verified manually by comparing the DOI, PMID where available, title, authors, journal, publication year, volume, and pagination. Fourteen confirmed duplicates were removed before title and abstract screening.

### 2.2. Inclusion Criteria

Studies were considered eligible for inclusion if they met the following criteria: (1) population consisting of adult patients with confirmed diagnoses of Crohn’s disease, ulcerative colitis, or IBD, broadly defined; (2) intervention involving biological agents or targeted small molecule therapies; (3) study design comprising randomised controlled trials (RCTs), phase 2 or phase 3 clinical trials, systematic reviews, network meta-analyses, or major narrative review articles; (4) outcomes reporting at least one of the following: clinical remission, endoscopic response or remission, safety profile, or pharmacological mechanism of action; and (5) publication between January 2021 and March 2026 in peer-reviewed journals, while landmark earlier trials and current guidelines were retained when necessary to contextualise established therapies.

### 2.3. Exclusion Criteria

Studies were excluded if they met any of the following criteria: (1) preclinical studies, including in vitro experiments and animal models; (2) publications prior to 2021, unless they represented landmark clinical evidence required to interpret an established therapy; (3) case reports, letters, editorials, or conference abstracts without full-text availability; (4) studies focusing exclusively on paediatric populations or IBD-related extraintestinal manifestations without reporting intestinal outcomes; and (5) articles without direct relevance to the biological or targeted pharmacological management of IBD.

### 2.4. Data Extraction

Two authors (A.O. and A.P.) independently extracted data from the included publications. Any discrepancies were resolved through discussion and consensus. Data were extracted independently and organised according to the following domains: (1) mechanism of action and therapeutic target; (2) clinical efficacy outcomes, including induction and maintenance remission rates from pivotal trials; (3) safety profile, encompassing rates of serious adverse events, infections, and class-specific risks; and (4) positioning in clinical practice, with attention to biologic-naive versus biologic-experienced populations and sequencing recommendations. Where network meta-analyses or comparative effectiveness studies were available, these were prioritised for cross-agent comparisons.

### 2.5. Study Selection

**Figure 1:**
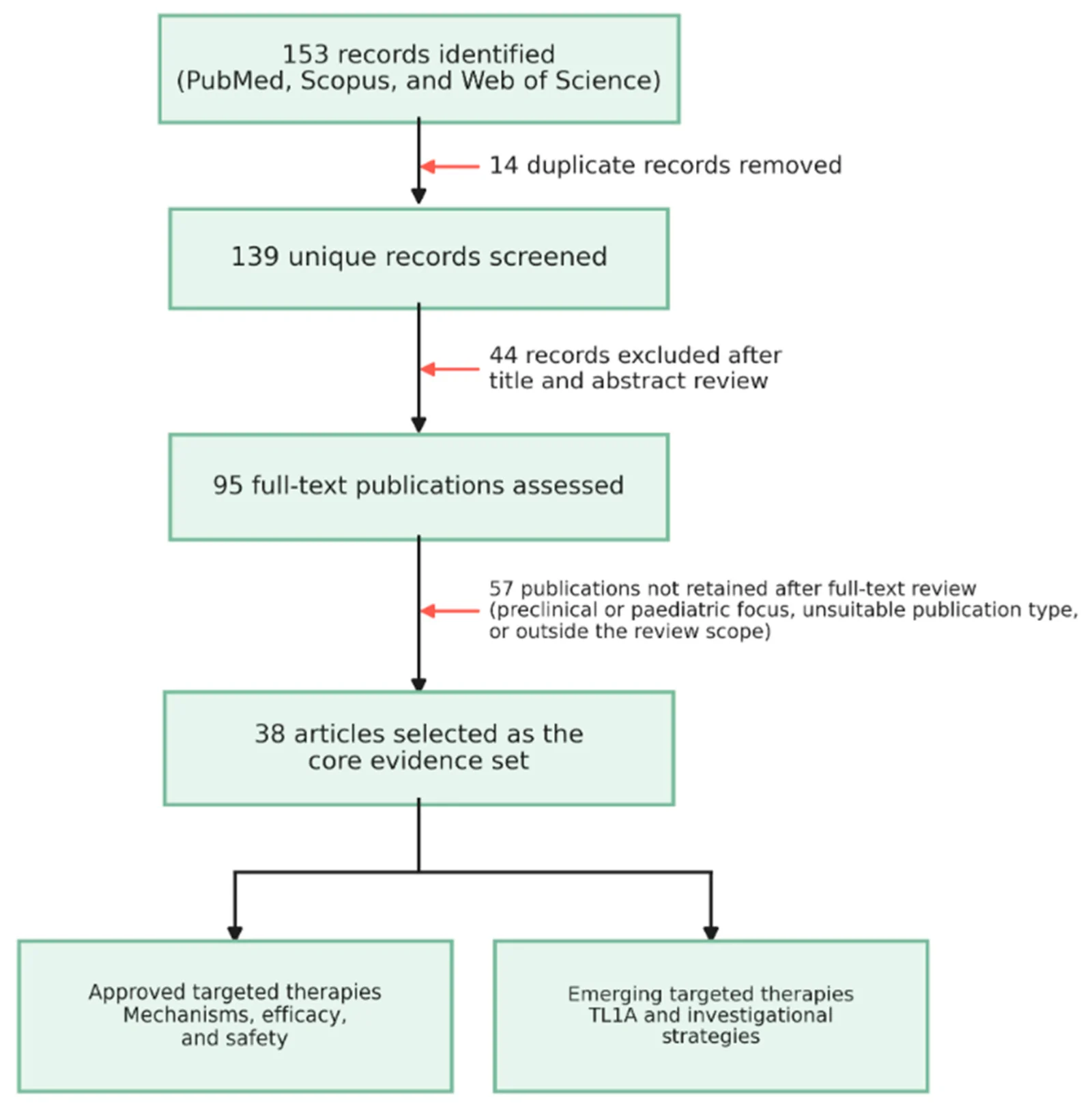
Structured overview of source identification and selection.

Two authors (A.O. and A.P.) independently screened the identified records by title and abstract and subsequently assessed potentially eligible publications in full text against the predefined inclusion and exclusion criteria. Any disagreement arising at either stage was resolved through discussion and consensus. The database searches identified 153 potentially relevant records. After the removal of 14 duplicates, 139 unique records underwent title and abstract review; 95 publications were subsequently examined in full text. Thirty-eight articles were selected as the core evidence set for the focused synthesis. Additional landmark trials and clinical guidelines were cited where required for historical or practice context. The structured source-selection overview is shown in Figure 1.

### 2.6. Evidence Synthesis and Critical Interpretation

Evidence was synthesised narratively according to the therapeutic mechanism, study design, disease indication, prior treatment exposure, and the maturity of the clinical development programme. Comparative statements were calibrated to the underlying evidence: direct randomised comparisons were separated from placebo-controlled efficacy estimates, network meta-analysis rankings, and observational effectiveness findings. No formal study-level risk-of-bias assessment or evidence-certainty grading was undertaken because the manuscript was designed as a focused narrative review rather than a systematic review.

Critical interpretation nevertheless considered recurrent design-specific limitations, including the selection of trial populations, heterogeneity in endpoint definitions and follow-up, sparse head-to-head evidence, transitivity assumptions in network meta-analyses, residual confounding and treatment-selection bias in observational cohorts, and the limited sample size and duration in early-phase studies. These considerations were used to moderate conclusions and are summarised qualitatively in Table 1.

## 3. Results

### 3.1. Pathophysiological Basis for Targeted Therapy

The pathogenesis of inflammatory bowel disease involves a complex and self-perpetuating interaction between genetic susceptibility, environmental triggers, the intestinal microbiome, and the mucosal immune system. In both Crohn’s disease and ulcerative colitis, a dysregulated innate and adaptive immune response leads to sustained inflammation of the intestinal wall, although the precise mechanisms, anatomical distribution, and dominant cytokine profiles differ between the two conditions. Understanding these pathways has been fundamental to the development of the biological and targeted therapies now available in clinical practice [1,2,4].

#### 3.1.1. Cytokine Networks and Inflammatory Cascades

Tumour necrosis factor-alpha (TNF-α) was the first cytokine identified as a central mediator of intestinal inflammation in IBD. TNF-α drives leukocyte recruitment, promotes epithelial apoptosis, and amplifies mucosal injury [1]. The IL-12/IL-23 axis is another critical pathway: IL-12 promotes T-helper 1 (Th1)-cell differentiation, while IL-23 sustains T-helper 17 (Th17)-cell survival and drives the production of IL-17 and IL-22, contributing to epithelial barrier dysfunction. IL-23—acting through its unique p19 subunit—has been identified as the dominant upstream regulator of intestinal inflammation in Crohn’s disease, providing the mechanistic rationale for selective IL-23p19 inhibitors [4]. The distinction between IL-12/IL-23 dual blockade and selective IL-23 inhibition has important clinical implications, as p19-selective agents preserve IL-12-mediated immune surveillance [4,8].

#### 3.1.2. Leukocyte Trafficking

A complementary mechanism driving intestinal inflammation is the selective recruitment of circulating lymphocytes to the gut mucosa, mediated by the interaction between the α4β7 integrin on lymphocyte surfaces and mucosal addressin cell adhesion molecule 1 (MAdCAM-1) on the intestinal endothelium. The blockade of this interaction reduces lymphocyte infiltration without broadly suppressing systemic immunity [16]. S1P receptor modulators interfere with lymphocyte egress from secondary lymphoid organs through a distinct but complementary mechanism [17,18].

#### 3.1.3. The JAK-STAT Signalling Pathway

Multiple pro-inflammatory cytokines—including IL-6, IL-12, IL-23, and interferons—exert their effects through JAK receptors coupled to STAT proteins. Because this pathway serves as a shared intracellular mediator for multiple cytokines simultaneously, its inhibition offers a broad anti-inflammatory effect with a single oral agent [2,9]. Janus kinase 1 (JAK1)-selective inhibitors—upadacitinib and filgotinib—have been developed to maximise efficacy while minimising off-target effects associated with pan-JAK inhibition [9].

#### 3.1.4. Rationale for Targeted Therapy

The identification of specific cytokines, adhesion molecules, and intracellular signalling nodes as drivers of intestinal inflammation has enabled the development of agents with increasing precision and selectivity [2,4]. These interventions act at three biological levels: extracellular cytokine blockade, control of leukocyte trafficking, and intracellular signal-transduction inhibition (Table 2). Mechanistic selectivity does not necessarily imply clinical superiority, and its value must be interpreted alongside indication, trial population, safety, and evidence maturity. Rather than broadly suppressing immune function, modern targeted therapies aim to interrupt defined pathological circuits while reserving protective immune responses, representing the conceptual foundation of contemporary IBD management [2].

### 3.2. Currently Approved Biologic Therapies

#### 3.2.1. Anti-TNF Agents

Anti-TNF agents, including infliximab, adalimumab, and golimumab, were the first biologic therapies approved for Crohn’s disease and ulcerative colitis and remain widely used due to their well-established efficacy and extensive long-term clinical experience. They remain important treatment options in severe Crohn’s disease, particularly in fistulising or penetrating phenotypes [2].

Infliximab has demonstrated sustained clinical and biochemical remission in real-world cohorts, although many patients require dose optimisation because of secondary loss of response [19]. Higher trough levels correlate with durable remission, while anti-drug antibodies are associated with treatment failure, supporting the established role of therapeutic drug monitoring [19]. Adalimumab is widely used because of its subcutaneous administration and favourable immunogenicity profile, and the baseline inflammatory burden may influence treatment response [1].

Network meta-analyses indicate that anti-TNF agents remain highly effective, particularly in biologic-naive patients, although newer agents such as IL-23 inhibitors or JAK inhibitors may offer superior outcomes in selected clinical scenarios [5,20]. Comparative cohort data indicate that serious-infection risk differs among biologic classes [13]. Golimumab, which is approved for UC, has demonstrated efficacy and an acceptable safety profile in comparative evidence [5]. Recent comparative evidence suggests that newer advanced therapies may provide advantages after prior biologic failure [5,7].

#### 3.2.2. Anti-Integrin Therapy

Vedolizumab has demonstrated consistent efficacy in both UC and CD, with a safety profile among the most favourable across advanced therapies. Indirect comparative evidence indicates that infliximab achieves higher induction efficacy than vedolizumab, but during maintenance, both agents achieve similar rates of remission and mucosal healing [16]. A large comparative meta-analysis showed ustekinumab achieves higher steroid-free remission than vedolizumab at post-induction (odds ratio [OR] 1.44; 95% confidence interval [CI] 1.11–1.88) and maintenance (OR 1.86; 95% CI 1.23–2.82) [21]. In biologic-naive CD, vedolizumab performs similarly to anti-TNF agents and ustekinumab, whereas in UC, differences between mechanisms become more pronounced [15]. Natalizumab has been nearly completely replaced by vedolizumab due to the risk of progressive multifocal leukoencephalopathy. In the direct head-to-head VARSITY trial, vedolizumab was superior to adalimumab for clinical remission and endoscopic improvement at week 52 in patients with ulcerative colitis, although superiority was not demonstrated for corticosteroid-free clinical remission [22].

#### 3.2.3. IL-12/IL-23 Inhibition—Ustekinumab

Ustekinumab, targeting the shared p40 subunit of IL-12 and IL-23, provides a balanced efficacy and safety profile. The 2024 meta-analysis in 2724 patients with prior anti-TNF failure confirmed superior steroid-free remission and clinical remission rates versus vedolizumab [21]. In ulcerative colitis, ustekinumab has demonstrated meaningful clinical and endoscopic benefits in the phase 3 UNIFI trial [23]. A 2025 network meta-analysis found that ustekinumab shows moderate but comparatively lower efficacy relative to newer p19-selective agents [8], with observational evidence supporting its use after previous biologic exposure [13,14].

#### 3.2.4. Selective IL-23 Inhibitors

Risankizumab was noninferior to ustekinumab for clinical remission at week 24 but it was superior for endoscopic remission at week 48 [24]. Network meta-analyses corroborate these findings, positioning selective IL-23 inhibitors among the most efficacious options for both the induction and maintenance of remission in CD, including in biologic-experienced patients [7].

### 3.3. Targeted Small Molecules

#### 3.3.1. JAK Inhibitors

Upadacitinib has shown a coherent and reproducible efficacy profile in Crohn’s disease. In the U-EXCEL and U-EXCEED induction trials, patients receiving 45 mg once daily achieved remission rates approaching half of the treated population (e.g., 49.5% versus 29.1% for clinical remission in U-EXCEL) [25]. Benefits translated into sustained disease control during U-ENDURE maintenance, with both 15 mg and 30 mg doses maintaining higher remission and endoscopic healing than placebo at week 52 [25]. A post hoc analysis confirmed efficacy regardless of prior biologic exposure [26], and long-term extension data confirmed durability over two years [27].

In ulcerative colitis, upadacitinib demonstrated strong performance across the U-ACHIEVE and U-ACCOMPLISH induction trials [28], with high sustained remission rates during U-ACHIEVE Maintenance [29]. Real-world data further confirm the effectiveness of upadacitinib reinduction and high-dose maintenance in clinical practice [30]. A propensity-matched cohort study found upadacitinib superior to tofacitinib in UC, with lower colectomy rates and fewer hospitalisations [31].

Filgotinib demonstrated efficacy in UC in the SELECTION program [32], and in CD, the DIVERSITY trial showed maintenance therapy achieving higher rates of clinical remission and endoscopic response than placebo (43.8% versus 26.4% at week 58) [33]. Tofacitinib requires careful patient selection given post-marketing data highlighting risks of venous thromboembolism and major adverse cardiovascular events in patients aged 50 years or older with cardiovascular risk factors [9]. In patients requiring a second JAK inhibitor after failure of the first, the J2J multicentre study showed acceptable effectiveness and safety [34].

#### 3.3.2. S1P Receptor Modulators

Ozanimod demonstrated clinical, endoscopic, and histologic benefits in the phase 3 True North trial, achieving higher rates of clinical remission than placebo during both induction (18.4% versus 6.0%) and maintenance (37.0% versus 18.5%) [17]. A comprehensive review further confirmed its favourable benefit–risk balance [10].

Etrasimod demonstrated robust efficacy in the ELEVATE programme: in ELEVATE UC 52, clinical remission was achieved in 27% at week 12 and maintained in 32% at week 52, versus 7% with the placebo at both time points [18]. A dedicated safety analysis showed similar infection rates between etrasimod and placebo, with no association between low lymphocyte counts and infection risk [35], supporting regulatory approval in both the United States and European Union [36]. A systematic review and meta-analysis of advanced oral small molecules confirmed the class-wide efficacy advantage of S1P modulators and JAK inhibitors over placebo, with distinct safety profiles warranting individualised patient selection [37].

### 3.4. Emerging Therapies

TL1A inhibition currently represents the most advanced emerging pathway under development. In a phase 2 trial in moderately to severely active UC, tulisokibart achieved higher rates of clinical remission and endoscopic improvement than placebo [38]. A companion phase 2a induction study in CD reported consistent trends [39]. Afimkibart demonstrated dose-dependent improvements in clinical, endoscopic, and histologic endpoints in the phase 2b TUSCANY-2 trial in UC [40]. Recent translational and pipeline reviews highlight TL1A inhibition as a potential next-generation cytokine axis, with the possibility of enabling more personalised treatment approaches based on TL1A expression and the genetic background [11,12].

At the molecular level, TL1A, encoded by tumour necrosis factor superfamily member 15 (TNFSF15), exerts its effects through death receptor 3 (DR3), also known as tumour necrosis factor receptor superfamily member 25 (TNFRSF25), thereby amplifying inflammatory signalling in activated T cells and other immune-cell populations. The persistent activation of the TL1A–DR3 pathway may also promote intestinal remodelling and fibrosis, making it relevant to both inflammatory activity and fibrostenotic disease phenotypes [11,12]. Mucosal TL1A expression, circulating TL1A concentrations, TNFSF15 genetic variants, and pathway-related transcriptional signatures have been investigated as potential biomarkers for treatment enrichment. However, none have yet been prospectively validated for routine therapeutic selection. Important translational challenges include assay standardisation, the identification of clinically meaningful biomarker thresholds, the confirmation of efficacy across ulcerative colitis and Crohn’s disease phenotypes, and the need for long-term safety, durability, and direct comparative data. Anti-TL1A therapy should therefore currently be regarded as an investigational strategy rather than an established biomarker-guided treatment approach.

Beyond TL1A, microbiome-based therapies, cell-based platforms, and novel small molecules targeting intracellular pathways—including next-generation JAK-selective or tyrosine kinase 2 (TYK2)-directed agents—represent an expanding area of early-phase research [2]. Collectively, these emerging therapies illustrate a rapidly evolving pipeline that may reshape future treatment paradigms in ulcerative colitis and Crohn’s disease. The comparative evidence, principal safety considerations, and practical positioning of the major approved and emerging therapeutic classes are summarised in Table 3.

## 4. Discussion

### 4.1. Evolution of IBD Treatment: From Generalised Immunosuppression to Targeted Therapy

The management of IBD has undergone a fundamental transformation over the past two decades. Anti-TNF agents marked the beginning of the biologic era, demonstrating that targeted cytokine blockade could alter the natural course of the disease. However, limitations including primary non-response, immunogenicity-driven secondary loss of response, and safety concerns requiring ongoing monitoring [3,13,19] have catalysed therapeutic innovation, leading to the approval of agents targeting integrin trafficking, IL-12/IL-23 and IL-23 pathways, JAK-STAT signalling, and sphingosine-1-phosphate receptors.

### 4.2. Advantages of Newer Therapies

In the SEQUENCE trial, risankizumab was noninferior to ustekinumab for week-24 clinical remission and superior for week-48 endoscopic remission [24]. Network meta-analyses and the 2025 AGA evidence synthesis further position selective IL-23 inhibitors among the higher-efficacy options for Crohn’s disease [8,42]. Upadacitinib has produced robust and durable remission rates across both CD and UC, including in biologic-experienced populations [25,26,27,28], confirmed by real-world data [30]. Gut-selective vedolizumab minimises systemic immunosuppression [15,16]; S1P modulators offer fully oral administration with reversible lymphocyte sequestration and a favourable infection profile [17,18,35], and JAK inhibitors provide the rapid onset of action and dose flexibility [25,28]. Newer therapies also demonstrate meaningful improvements in patient-reported outcomes and quality of life [37,42,43,44].

### 4.3. Current Challenges

The absence of validated predictive biomarkers continues to impede personalised therapy selection [2]. Cost and healthcare system access represent a major barrier, particularly for the newest agents, although biosimilar availability has reduced costs for established biologics [5]. Long-term safety data for several newer agents remain limited, and post-marketing surveillance will be essential to characterise rarer adverse events [9,13]. The feasibility and safety of sequential JAK inhibitor use have begun to be characterised by the J2J study [34]. The management of difficult-to-treat IBD in patients who have failed multiple lines of advanced therapy remains an area of substantial unmet need [14].

### 4.4. Therapy Sequencing

Current AGA guidelines recommend multiple advanced therapies for moderate-to-severe IBD, with treatment selection informed by disease phenotype, previous treatment exposure, comparative efficacy, and safety [45,46]. Anti-TNF agents remain particularly relevant in severe or fistulising Crohn’s disease [46]. Vedolizumab and ustekinumab represent well-evidenced alternatives when safety is a priority [15,16,41]. Following anti-TNF failure, ustekinumab has demonstrated consistently superior outcomes over vedolizumab in CD [21], while selective IL-23 inhibitors and JAK inhibitors represent the most evidence-based options in both anti-TNF-naive and biologic-experienced patients [5,7,8,24,42]. In UC, upadacitinib and etrasimod have emerged as robust options after anti-TNF failure [18,28,29], and the AGA UC guideline provides structured sequencing recommendations integrating disease activity, prior exposure, and patient risk factors [43,45]. The distinction between CD and UC remains clinically meaningful, as agents that perform comparably in one condition may diverge substantially in the other [15].

### 4.5. Future Perspectives

**Figure 2:**
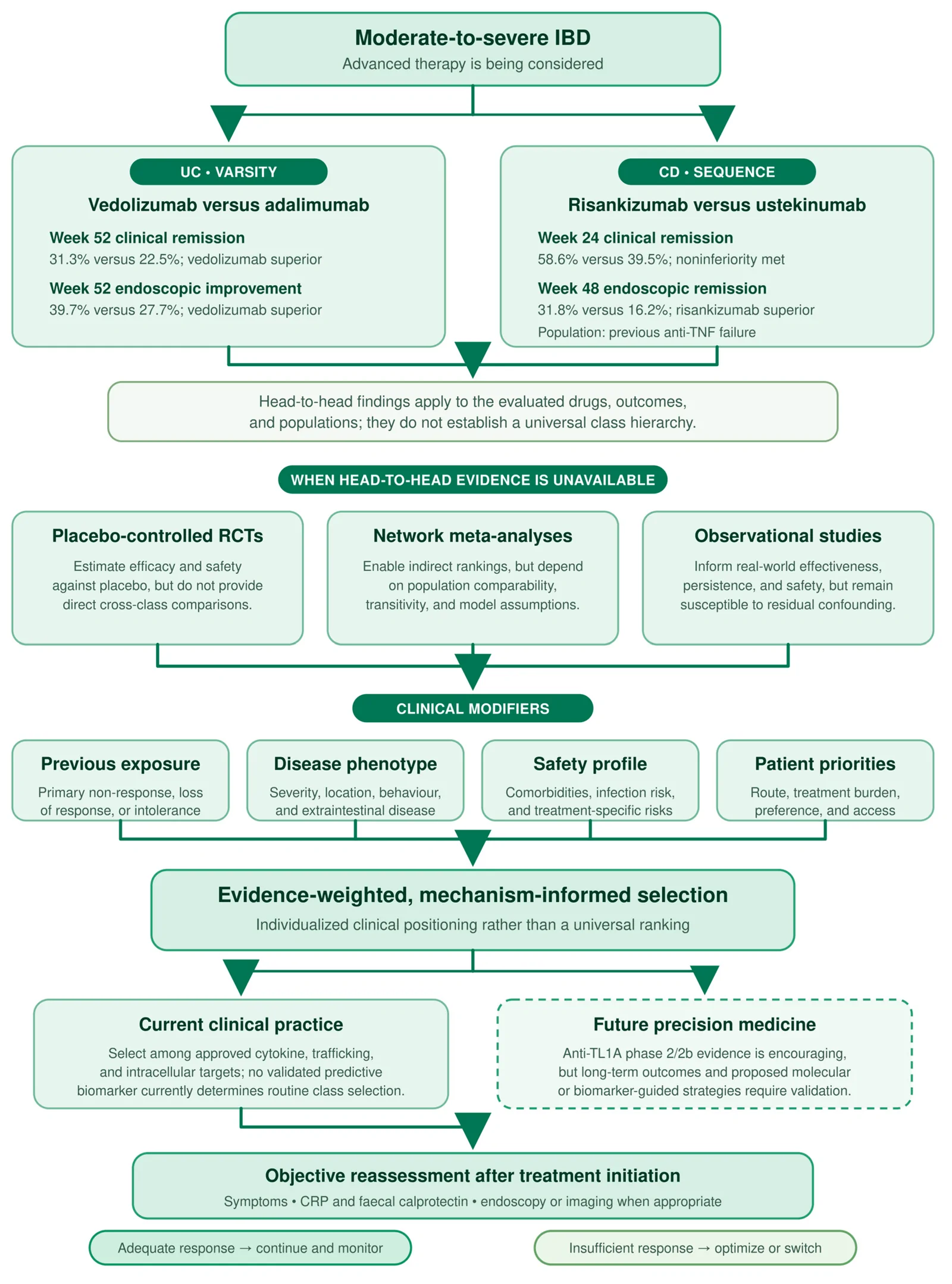
Evidence-informed framework for positioning advanced therapies in inflammatory bowel disease. Direct evidence from the VARSITY and SEQUENCE trials is distinguished from placebo-controlled trials, network meta-analyses, and observational evidence. Treatment selection should integrate disease phenotype, previous treatment exposure, safety considerations, and patient priorities. Anti-TL1A and biomarker-guided strategies remain investigational. Abbreviations: anti-TNF, anti–tumour necrosis factor; CD, Crohn’s disease; CRP, C-reactive protein; IBD, inflammatory bowel disease; RCTs, randomised controlled trials; TL1A, tumour necrosis factor-like cytokine 1A; UC, ulcerative colitis.

Anti-TL1A agents—tulisokibart and afimkibart—represent the most advanced emerging class, with phase 2 and 2b data demonstrating meaningful clinical, endoscopic, and histologic benefits [11,12,38,39,40]. Comparative-effectiveness evidence and the prospective validation of candidate biomarkers remain necessary before biomarker-guided treatment selection can be incorporated into routine clinical practice [11,12,42,46]. Dual-targeted therapy combining two advanced agents with complementary mechanisms is under active investigation and may offer a strategy for overcoming the therapeutic ceiling in refractory disease. Cellular and regenerative therapies, including mesenchymal stromal cell platforms and microbiome-based interventions, represent exploratory but potentially transformative directions [2]. The relationship between comparative evidence, clinical modifiers, current therapeutic positioning, and emerging precision-medicine strategies is summarised in Figure 2.

### 4.6. Limitations

Several limitations should be considered when interpreting this review. First, the principal literature search covered the period from January 2021 to March 2026 and produced a focused core evidence set of 38 publications. Landmark trials published before 2021 and relevant clinical guidelines were retained where necessary, but some earlier or non-indexed studies may nevertheless have been missed. Second, this article was designed as a focused narrative review. The structured search was intended to improve transparency rather than provide exhaustive systematic coverage. Accordingly, methodological appraisal focused on the limitations associated with each source of evidence rather than formal study-level risk-of-bias scoring, and no quantitative pooling was performed. Third, the available studies differed considerably in disease phenotype, previous exposure to advanced therapies, outcome definitions, follow-up duration, and study design, limiting direct comparability. Only a small number of head-to-head randomised trials are available; therefore, broader cross-class conclusions rely mainly on placebo-controlled trials, network meta-analyses, and observational cohorts. Network estimates depend on assumptions of transitivity and consistency, whereas observational findings remain susceptible to residual confounding and treatment-selection bias. Finally, evidence for anti-TL1A agents is derived mainly from phase 2 and 2b trials. Their comparative effectiveness, long-term safety, durability of response, and potential role in biomarker-guided treatment selection remain uncertain. These limitations support the cautious interpretation of therapeutic rankings and highlight the need for additional direct comparisons and prospective biomarker-validation studies.

## 5. Conclusions

This review synthesises the current evidence on biological and targeted therapies for inflammatory bowel disease, encompassing approved agents and an emerging therapeutic pipeline. The available literature supports a paradigm shift from generalised immunosuppression toward mechanism-specific, patient-tailored treatment strategies. Anti-TNF agents retain a central role, particularly in biologic-naive patients, while newer classes—selective IL-23 inhibitors, JAK inhibitors, and S1P modulators—have demonstrated robust efficacy and distinct safety profiles. Anti-TL1A inhibition represents a compelling next frontier, with early clinical data suggesting meaningful potential in both CD and UC.

Nonetheless, key challenges persist: the absence of validated predictive biomarkers, limited long-term safety data for newer agents, and the complexity of optimal therapy sequencing in biologic-experienced patients. Addressing these gaps through prospective biomarker studies, long-term registries, and head-to-head comparative trials will be essential. Future treatment algorithms in IBD are likely to be defined by precision medicine principles, integrating molecular profiling, disease phenotypes, and individual patient risk factors to guide rational and personalised therapeutic decisions.

## Figures and Tables

**Table 1:** Evidence sources and interpretive limitations in inflammatory bowel disease.

Evidence Source	Principal Contribution	Main Interpretive Limitation
Head-to-head randomised trials	Direct comparison between active treatments	Few available comparisons; findings remain population- and endpoint-specific
Placebo-controlled randomised trials	Efficacy and safety relative to placebo	Limited cross-class inference and highly selected study populations
Network meta-analyses	Comparative estimates across multiple therapies	Dependence on transitivity, consistency, endpoint alignment, and heterogeneity
Observational cohorts	Real-world effectiveness and safety	Residual confounding, treatment selection, missing data, and variable follow-up
Early-phase studies	Preliminary efficacy, dose-response, and mechanistic signals	Small samples and limited durability, rare-harm, and comparative data

**Note:** This table summarises the main contribution and common interpretive limitations of each evidence source discussed in this narrative review. It is intended as a qualitative framework and does not represent a formal risk-of-bias assessment or grading of evidence certainty. Limitations may vary across individual studies.

**Table 2:** Mechanism-based therapeutic landscape of approved and emerging targeted therapies in inflammatory bowel disease.

Biological Level	Target/Pathway	Representative Agents
Extracellular cytokines	TNF-α IL-12/IL-23 p40 IL-23 p19 TL1A-DR3	Infliximab, adalimumab Ustekinumab Risankizumab, mirikizumab Tulisokibart, afimkibart *
Leukocyte trafficking	α4β7-MAdCAM-1 S1P receptor signalling	Vedolizumab Ozanimod, etrasimod
Intracellular signalling	JAK-STAT cascade	Tofacitinib Upadacitinib, filgotinib

**Note:** Therapeutic classes are organised by biological level, and representative agents are illustrative rather than exhaustive. * Anti-TL1A agents remain investigational; approved indications for other agents differ between Crohn’s disease and ulcerative colitis.

**Table 3:** Comparative clinical overview of approved and emerging advanced therapies for inflammatory bowel disease.

Therapeutic Class and Representative Agents	IBD Setting	Key Evidence and Efficacy	Main Safety Considerations	Practical Clinical Positioning
**Anti-TNF agents** **Infliximab,** **adalimumab;** **golimumab (UC)**	CD and UC	Extensive randomised and real-world evidence supports induction and maintenance efficacy, with an established role in fistulising CD. Network evidence remains favourable in biologic-naive patients [5,19,20].	Serious or opportunistic infection; tuberculosis and hepatitis B reactivation; immunogenicity; infusion or injection reactions.	Established option, particularly for severe or fistulising CD; extensive long-term experience, therapeutic drug monitoring, and biosimilar availability.
**Anti-integrin** **therapy** **Vedolizumab**	CD and UC	VARSITY showed higher week-52 clinical remission and endoscopic improvement with vedolizumab than adalimumab in UC. Cohort and indirect evidence supports effectiveness in CD and UC [6,15,16,21,22].	Generally favourable gut-selective profile; infection and infusion-reaction monitoring remain appropriate.	Useful when safety is prioritised, including in older or comorbid patients; direct comparative evidence supports its use in UC.
**IL-12/IL-23** **p40 inhibition** **Ustekinumab**	CD and UC	UNIFI established efficacy in UC, while SEAVUE showed efficacy comparable to adalimumab in biologic-naive CD. Post-anti-TNF comparative evidence supports effectiveness [15,21,23,41].	Generally favourable safety profile; monitor infection risk and treatment-emergent adverse events.	Balanced efficacy and safety option in CD and UC; useful after anti-TNF failure or when convenient maintenance is preferred.
**Selective IL-23** **p19 inhibition** **Risankizumab,** **mirikizumab**	CD and UC; agent-specific	In SEQUENCE, risankizumab was noninferior to ustekinumab for week-24 clinical remission and superior for week-48 endoscopic remission after anti-TNF failure. Network evidence places selective IL-23 agents among higher-efficacy options [7,8,24,42].	Favourable short-term profile; monitor infections and agent-specific laboratory abnormalities. Long-term comparative safety continues to evolve.	High-efficacy option in CD and UC, including biologic-experienced disease; the strongest direct evidence currently applies to risankizumab in CD.
**JAK inhibition** **Tofacitinib,** **upadacitinib,** **filgotinib**	UC and CD; agent-specific	Upadacitinib showed robust induction and maintenance efficacy in UC and CD (U-ACHIEVE/U-ACCOMPLISH and U-EXCEL/U-EXCEED/U-ENDURE). Tofacitinib and filgotinib have agent-specific placebo-controlled and real-world evidence [25,26,27,28,29,30,31,32,33,34].	Serious infection and herpes zoster; laboratory abnormalities; regulatory warnings concerning MACE, venous thromboembolism, and malignancy in higher-risk populations.	Rapid-onset oral option, often considered after biologic failure or when rapid control is required; careful risk stratification is essential.
**S1P receptor** **modulation** **Ozanimod,** **etrasimod**	UC	True North and ELEVATE UC 12/52 demonstrated induction and maintenance efficacy over placebo in UC; no direct cross-class randomised comparison is available [10,17,18,35,36,37].	Bradycardia or conduction effects, macular oedema, lymphopenia, elevated liver enzymes, and infections; pretreatment assessment is agent-specific.	Oral UC option for patients preferring non-injectable therapy; consider cardiac, ophthalmic, hepatic, and interaction-related factors.
**Anti-TL1A** **therapy** **(investigational)** **Tulisokibart,** **afimkibart**	UC and CD clinical trials	Phase 2 and 2b studies reported clinical and endoscopic activity for tulisokibart and afimkibart in UC, with early CD evidence for tulisokibart. Comparative and long-term data remain unavailable [11,12,38,39,40].	Exposure remains limited to early-phase trials; uncommon and long-term adverse events remain uncertain.	Clinical-trial use only. A potential future role in biomarker-guided treatment selection remains unvalidated.

**Note:** Therapeutic positioning is intended as a concise evidence-based overview rather than a prescriptive treatment algorithm. The IBD setting reflects disease settings represented in the reviewed evidence and does not imply identical regulatory approval across agents or regions. Abbreviations: anti-TNF, anti–tumour necrosis factor; CD, Crohn’s disease; IBD, inflammatory bowel disease; IL, interleukin; JAK, Janus kinase; MACE, major adverse cardiovascular events; S1P, sphingosine-1-phosphate; TL1A, tumour necrosis factor-like cytokine 1A; UC, ulcerative colitis.

## Data Availability

No new data were created or analysed in this study. Data sharing is not applicable to this article.

## Supplementary Materials

The following supporting information can be downloaded at: https://www.mdpi.com/article/10.3390/jcm15176753/s1, Table S1: Complete database-specific search strategies and deduplication procedure.

## Abbreviations

The following abbreviations are used in this manuscript:

AGA	American Gastroenterological Association
anti-TNF	anti–tumour necrosis factor
CD	Crohn’s disease
CI	confidence interval
CRP	C-reactive protein
DR3	death receptor 3
IBD	inflammatory bowel disease
IL	interleukin
JAK	Janus kinase
JAK1	Janus kinase 1
MACE	major adverse cardiovascular events
MAdCAM-1	mucosal addressin cell adhesion molecule 1
MeSH	Medical Subject Headings
OR	odds ratio
RCT	randomised controlled trial
S1P	sphingosine-1-phosphate
STAT	signal transducer and activator of transcription
Th1	T helper 1
Th17	T helper 17
TL1A	tumour necrosis factor-like cytokine 1A
TNF-α	tumour necrosis factor alpha
TNFRSF25	tumour necrosis factor receptor superfamily member 25
TNFSF15	tumour necrosis factor superfamily member 15
TYK2	tyrosine kinase 2
UC	ulcerative colitis
