# Supplementary material for: Targeted Therapies in Inflammatory Bowel Disease: Mechanisms, Comparative Evidence, and Clinical Translation
Source: J Clin Med. 2026 Aug 31;15(17):6753. doi: 10.3390/jcm15176753 (PMC13566816; doi:10.3390/jcm15176753)
Supplement: Supplementary file 1 [file jcm-15-06753-s001.zip › jcm-4477873-supplementary.pdf]

Supplementary Material

Targeted Therapies in Inflammatory Bowel Disease: Mechanisms, Comparative Evidence, and Clinical Translation

Table S1. Complete database-specific search strategies and deduplication procedure.

Database	Search fields	Complete Boolean search strategy	Limits and final search date
**PubMed**	MeSH Terms; Title/Abstract; Publication Date	(("Inflammatory Bowel Diseases"[MeSH Terms] OR "Crohn Disease"[MeSH Terms] OR "Colitis, Ulcerative"[MeSH Terms] OR "inflammatory bowel disease"[Title/Abstract] OR "Crohn disease"[Title/Abstract] OR "Crohn's disease"[Title/Abstract] OR "ulcerative colitis"[Title/Abstract])<br>AND<br>("Biological Therapy"[MeSH Terms] OR biologic*[Title/Abstract] OR "biological therapy"[Title/Abstract] OR "biological therapies"[Title/Abstract] OR "targeted therapy"[Title/Abstract] OR "targeted therapies"[Title/Abstract] OR "small molecule"[Title/Abstract] OR "small molecules"[Title/Abstract] OR "Janus kinase inhibitor"[Title/Abstract] OR "Janus kinase inhibitors"[Title/Abstract] OR "JAK inhibitor"[Title/Abstract] OR "JAK inhibitors"[Title/Abstract] OR "interleukin-23 inhibitor"[Title/Abstract] OR "interleukin-23 inhibitors"[Title/Abstract] OR "IL-23 inhibitor"[Title/Abstract] OR "IL-23 inhibitors"[Title/Abstract] OR "anti-TNF"[Title/Abstract] OR vedolizumab[Title/Abstract] OR ustekinumab[Title/Abstract] OR risankizumab[Title/Abstract] OR mirikizumab[Title/Abstract] OR tofacitinib[Title/Abstract] OR upadacitinib[Title/Abstract] OR filgotinib[Title/Abstract] OR ozanimod[Title/Abstract] OR etrasimod[Title/Abstract] OR "sphingosine-1-phosphate receptor modulator"[Title/Abstract] OR "sphingosine-1-phosphate receptor modulators"[Title/Abstract] OR "S1P receptor modulator"[Title/Abstract] OR "S1P receptor modulators"[Title/Abstract] OR TL1A[Title/Abstract] OR tulisokibart[Title/Abstract] OR afimkibart[Title/Abstract]))<br>AND<br>("2021/01/01"[Date - Publication] : "2026/03/31"[Date - Publication])	Publication years: 2021-2026<br>No language restriction<br>Final search: 31 March 2026
**Scopus**	Article title, abstract, and keywords (TITLE-ABS-KEY)	TITLE-ABS-KEY(("inflammatory bowel disease" OR "Crohn disease" OR "Crohn's disease" OR "ulcerative colitis")<br>AND<br>("biological therapy" OR "biological therapies" OR biologic* OR "targeted therapy" OR "targeted therapies" OR "small molecule" OR "small molecules" OR "Janus kinase inhibitor" OR "Janus kinase inhibitors" OR "JAK inhibitor" OR "JAK inhibitors" OR "interleukin-23 inhibitor" OR "interleukin-23 inhibitors" OR "IL-23 inhibitor" OR "IL-23 inhibitors" OR "anti-TNF" OR vedolizumab OR ustekinumab OR risankizumab OR mirikizumab OR tofacitinib OR upadacitinib OR filgotinib OR ozanimod OR etrasimod OR "sphingosine-1-phosphate receptor modulator" OR "sphingosine-1-phosphate receptor modulators" OR "S1P receptor modulator" OR "S1P receptor modulators" OR TL1A OR tulisokibart OR afimkibart))<br>AND PUBYEAR > 2020 AND PUBYEAR < 2027	Publication years: 2021-2026<br>No language restriction<br>Final search: 31 March 2026
**Web of Science Core Collection**	Topic (TS): title, abstract, author keywords, and Keywords Plus	TS=(("inflammatory bowel disease" OR "Crohn disease" OR "Crohn's disease" OR "ulcerative colitis")<br>AND<br>("biological therapy" OR "biological therapies" OR biologic* OR "targeted therapy" OR "targeted therapies" OR "small molecule" OR "small molecules" OR "Janus kinase inhibitor" OR "Janus kinase inhibitors" OR "JAK inhibitor" OR "JAK inhibitors" OR "interleukin-23 inhibitor" OR "interleukin-23 inhibitors" OR "IL-23 inhibitor" OR "IL-23 inhibitors" OR "anti-TNF" OR vedolizumab OR ustekinumab OR risankizumab OR mirikizumab OR tofacitinib OR upadacitinib OR filgotinib OR ozanimod OR etrasimod OR "sphingosine-1-phosphate receptor modulator" OR "sphingosine-1-phosphate receptor modulators" OR "S1P receptor modulator" OR "S1P receptor modulators" OR TL1A OR tulisokibart OR afimkibart))<br>AND PY=(2021-2026)	Publication years: 2021-2026<br>No language restriction<br>Final search: 31 March 2026

Deduplication procedure. All records retrieved from the three databases were imported into Rayyan. Potential duplicates were flagged using Rayyan's duplicate-detection function and were subsequently verified manually by comparing digital object identifier (DOI), PubMed identifier (PMID) where available, title, authors, journal, publication year, volume, and pagination (A.O. and A.P.). Fourteen confirmed duplicates were removed, leaving 139 unique records for title and abstract screening.

Note: The principal database search covered publications from 1 January 2021 to 31 March 2026. Earlier landmark trials and current clinical guidelines were retained when necessary to provide historical or practice context.
